# Brain and effort: brain activation and effort-related working memory in healthy participants and patients with working memory deficits

**DOI:** 10.3389/fnhum.2013.00140

**Published:** 2013-04-17

**Authors:** Maria Engström, Anne-Marie Landtblom, Thomas Karlsson

**Affiliations:** ^1^Radiology, Department of Medical and Health Sciences, Linköping UniversityLinköping, Sweden; ^2^Center for Medical Image Science and Visualization (CMIV), Linköping UniversityLinköping, Sweden; ^3^Neurology, Department of Clinical and Experimental Medicine, Linköping University and UHL, County CouncilLinköping, Sweden; ^4^Disability Research, Department of Behavioral Sciences and Learning, Linköping UniversityLinköping, Sweden; ^5^Linnaeus Centre HEAD, Linköping UniversityLinköping, Sweden

**Keywords:** functional magnetic resonance imaging (fMRI), executive network, salience network, complex working memory, working memory deficits, periodic idiopathic hypersomnia, Kleine–Levin syndrome (KLS)

## Abstract

Despite the interest in the neuroimaging of working memory, little is still known about the neurobiology of complex working memory in tasks that require simultaneous manipulation and storage of information. In addition to the central executive network, we assumed that the recently described salience network [involving the anterior insular cortex (AIC) and the anterior cingulate cortex (ACC)] might be of particular importance to working memory tasks that require complex, effortful processing.

**Method:** Healthy participants (*n* = 26) and participants suffering from working memory problems related to the Kleine–Levin syndrome (KLS) (a specific form of periodic idiopathic hypersomnia; *n* = 18) participated in the study. Participants were further divided into a high- and low-capacity group, according to performance on a working memory task (listening span). In a functional magnetic resonance imaging (fMRI) study, participants were administered the reading span complex working memory task tapping cognitive effort.

**Principal findings:** The fMRI-derived blood oxygen level dependent (BOLD) signal was modulated by (1) effort in both the central executive and the salience network and (2) capacity in the salience network in that high performers evidenced a weaker BOLD signal than low performers. In the salience network there was a dichotomy between the left and the right hemisphere; the right hemisphere elicited a steeper increase of the BOLD signal as a function of increasing effort. There was also a stronger functional connectivity within the central executive network because of increased task difficulty.

**Conclusion:** The ability to allocate cognitive effort in complex working memory is contingent upon focused resources in the executive and in particular the salience network. Individual capacity during the complex working memory task is related to activity in the salience (but not the executive) network so that high-capacity participants evidence a lower signal and possibly hence a larger dynamic response.

## Introduction

Working memory involves the temporary storage and manipulation of information for the purpose of the analysis, production, and communication of ideas and intentions (Miller et al., 1960; Baddeley and Hitch, 1974; Miyake and Shah, 1999). Hence, working memory capacity is a key determinant to reading, language comprehension, intelligence, and many other activities involving reasoning or planning for the future (Kyllonen and Christal, 1990; Engle et al., 1999; Conway et al., 2002; Ackerman et al., 2005; Oberauer et al., 2008). Indeed, some authors have claimed that intelligence and problem-solving skills are more or less synonymous with complex working memory (Kyllonen and Christal, 1990; Engle et al., 1999).

Although working memory is central to our current understanding of cognitive networks residing in the brain, a large majority of neuroimaging experiments have used tasks that tap upon automatized aspects of working memory (such as the digit span task and the Sperling whole/partial report method) rather than complex uses of working memory. While these short-term memory tasks address fundamental properties of working memory (e.g., temporary storage of information or online representation of phonological information), performance on these low-level working memory measures is weakly related to language comprehension, reasoning, and problem solving skills in both academic and real-world settings (Daneman and Carpenter, 1980; Kyllonen and Christal, 1990; Shute, 1991). In contrast, tasks that require the simultaneous storage and manipulation of information evidence a stronger association with activities related to thinking and problem solving (Oberauer et al., 2008; Duncan et al., 2012). The reason why complex working memory tasks more than traditional short-term memory tasks are related to fundamental aspects of cognition is that complex tasks involve larger investments of attentional resources (Unsworth and Engle, 2007). Traditional short-term tasks, on the other hand, capitalize relatively more on skills that have become automatized through extended practice (e.g., rehearsal of digits, which is an integral part of many everyday situations, including simple arithmetic tasks) and skills that involve highly dedicated neural systems (e.g., the phonological loop, endowing brief, online access to phonological information). Thus, a fundamental difference between traditional (short-term memory) and complex working memory tasks is that the latter require the investment of cognitive effort to a larger degree than the former.

Cognitive theories place special emphasis upon the effortful allocation of cognitive resources (Pribram and McGuinness, 1975, 1992) through a global workspace or a central executive (Dehaene et al., 1998; Baddeley, 2003; Baars, 2005). The concept of effort was re-introduced in cognitive psychology by Kahneman (1973). According to Kahneman, cognitive processes differ in attentional requirements. Tasks—such as working memory tasks—vary along a continuum from highly automatized to highly effortful tasks. Moreover, individuals differ in terms of the pool of available attentional resources. Despite the importance of effortful processing in our understanding of complex working memory, a mere handful of neuroimaging studies have examined the role of cognitive effort in working memory (Jansma et al., 2007; Lim et al., 2010; Chein et al., 2011; Demeter et al., 2011). A main finding in these studies has been that the middle and inferior prefrontal gyri, the inferior parietal cortex, and the thalamus are sensitive to attentional demands.

Although neuroimaging studies of effortful processing in working memory remain scarce, neuropsychological studies of patients with circumscribed brain lesions have explicitly implicated the anterior cingulate cortex (ACC) in relation to effortful processing (Mulert et al., 2005; Kohl et al., 2009). These results fit well with clinical observations from individual cases with focal lesions to the ACC: lack of motivation, spontaneity, and willpower (Damasio and Van Hoesen, 1983; Cohen et al., 1999). At the same time, Fellows and Farah failed to document any impairment at all with respect to cognitive control in four patients with ACC damage due to stroke involving the territory of the anterior cerebral artery (Fellows and Farah, 2005). Hence, it would be illuminating to study the involvement of the ACC and related structures in cognitive effort by means of functional magnetic resonance imaging (fMRI).

The crucial role of effortful processing has been the focus of interest in hypersomnia, a cluster of neurological disorders characterized by recurrent sleep attacks of varying duration. In our laboratory, we have recently described a selective impairment of complex working memory in patients suffering from the Kleine–Levin syndrome (KLS). In addition to repeated episodes of prolonged sleep periods (lasting from days to weeks), these patients also present with hyperphagia, cognitive impairment, and behavioral disturbances during attacks. KLS is characterized by marked and persistent problems with regard to complex working memory dysfunction between episodes and sometimes even after the hypersomnia has receded (Landtblom et al., 2002, 2003; Engström et al., 2009). These problems involving working memory and attention take place in the context of preserved general cognitive capacity and—which is of particular importance—intact performance on less demanding short-term memory tasks, such as repetition of digits and letters (Engström et al., 2009).

In a recent fMRI study, we found that brain activation in areas related to working memory and effortful processing increased significantly as a function of mounting effort in the reading span task (Engström et al., 2009). We also showed decreased activation in the ACC and the anterior insular cortex (AIC) and increased activation in the left dorsolateral prefrontal cortex (DLPFC) as well as more bilateral activity in posterior parietal cortex (PPC) in KLS. In addition, we observed hyperactivity in the left thalamus in KLS but not in healthy participants. The altered brain activation pattern in KLS patients was accompanied by lower performance levels and longer reaction times.

In addition to functional differences between KLS and controls, we have also observed that the level of activation differs between individuals with low and high working memory capacity. In a study involving young, healthy participants, performance on the reading span task (administered during a neuropsychological screening session, taking place before the fMRI session) predicted activation during a working memory task performed during scanning in that high working memory capacity participants evidenced lower activity (in technical terms, a lower intercept) especially when load was low (Engström et al., 2012). In other words, it appears as if high performers started from a lower baseline and then modulated processing along a larger continuum. This pattern is similar to the notion of neural efficiency, as proposed by Neubauer and Fink (2009). These investigators suggested that brighter individuals display more efficient (i.e., lower) brain activation during processing. Although the hypothesis originally was invoked to explain performance on tasks related to intelligence (and not working memory), it bears a clear resemblance to our empirical pattern. Hence, in addition to the prediction of differences between KLS and controls in terms of response to working memory load, and high scorers vs. low scorers, we also hypothesized that high scorers would take off from a lower level than low scorers and thus modulate the blood oxygen level dependent (BOLD) response to effort along a larger dynamic range. We were also interested to find out if this putative pattern appears in all brain regions critical to attention and effort, or if it characterized by regional specificity.

The main purpose of this communication was therefore to clarify how brain activity is modulated by cognitive effort and individual capacity in a complex working memory task. Given the lead by results from Jansma et al. (2007) and Osaka and co-workers (Osaka et al., 2003, 2004), we propose that complex working memory reflects activity in the “classical” working memory system, typically involving DLPFC and PPC (Cabeza and Nyberg, 2000; Cole and Schneider, 2007) plus activity in the AIC, ACC, and the thalamus.

The advent of new neuroimaging modalities has revitalized the interest in the network properties of brain systems (Greicius et al., 2003; Fox et al., 2005; Bullmore and Sporns, 2009; Bressler and Menon, 2010; Graham and Rockmore, 2011). Despite a large number of investigations involving automatic or storage aspects of working memory [see Cabeza and Nyberg (2000) and Wager and Smith (2003); and references therein], the delineation of the network (Rubinov and Sporns, 2010) of complex working memory has only recently begun (Kondo et al., 2004; Ginestet and Simmons, 2011). Working memory studies (of which a large majority involves low-level aspects of working memory) highlight dorsolateral prefrontal and inferior parietal areas in working memory, that is to say the executive network (Collette and Van der Linden, 2002). Studies involving mental effort implicate large portions of the proisocortex, the AIC, the ACC, and the thalamus in recruitment of cognitive resources. These areas constitute nodes of a presumptive network that recently was termed the salience network (Downar et al., 2002; Seeley et al., 2007; Menon and Uddin, 2010), since one of the main functions of this structure is the detection of the relevance of external and bodily events [Sadaghiani et al. (2010) for a topical and somewhat different interpretation]. However, other investigators have used other terms to label this network, such as the tonic alertness network (Sturm and Willmes, 2001; Sadaghiani et al., 2010) or task set maintenance network (Dosenbach et al., 2007) and yet other researchers include nodes in more widespread temporo-parietal-frontal areas to be part of the salience network (Downar et al., 2002). In the present communication, we define the salience network in terms of the AIC, ACC, and the thalamus.

More explicitly, a second purpose of this study was to investigate if the interactions within and between the executive and salience networks are changed in a more effortful condition of the working memory task and if these interactions are different in individuals with different cognitive capacity. We did so by comparing brain activation at different levels of difficulty of the complex working memory (reading span) task. We also did so by means of a comparison between healthy participants and participants suffering from KLS. We assumed that increasing effort while keeping task requirements constant would indicate a more coherent coupling within the executive and salience networks, respectively. We also predicted less coherent network connectivity in KLS, indicating difficulties in assembling neural resources in the more demanding version of the task. Casted in terms of network architecture, we assumed that cognitive effort would involve both the executive and salience networks. More importantly, we assumed that differences between high- and low performers would be marked in the salience network, given that the constituents of this network—the ACC, AIC, and the thalamus—are critical to cognitive control and adjustment of effortful processing.

## Materials and methods

### Participants

Twenty-six healthy participants were recruited to the study (mean age = 24.1 years, *SD* = 5.3 years, females/males = 14/12). According to clinical interviews the participants had no known medical, psychiatric, or neurological disorder (including sleep disorder), nor cognitive dysfunction. The participants were also screened for language ability to control for proper execution of the verbal tasks. Two participants were excluded due to inadequate language proficiency and they were replaced with native subjects. One non-native Swedish speaker was retained in the study. A subgroup of 18 participants, matched for age and sex with patients with KLS, was selected as a control group. The mean age of the controls were 24.7 years (*SD* = 6.1 years).

Eighteen patients with KLS were consecutively recruited to the study from the Department of Neurology at Linköping University Hospital, which receives patients from Nordic countries with suspected KLS. Inclusion criterion was fulfilled ICSD-2 criteria for KLS (American Academy of Sleep Medicine, 2005). Exclusion criteria were secondary KLS caused by trauma or other brain lesion and contraindications for MRI. All patients were examined when they were in an asymptomatic state, i.e., during the interval between sleep episodes or after the last relapse. One patient was treated with a serotonin reuptake inhibitor. No patient received analeptics at the time of the study. The mean age of the KLS patients was 25.9 years (*SD* = 11.4 years). Ten patients were females and 8 males. Patients in our study have a larger female to male ratio compared to other studies, which have found KLS to be more prevalent in males. Three patients had Norwegian as their first language, but had satisfactory comprehension of Swedish as assessed by the language-screening test. The mean age of onset was 15.5 years (*SD* = 1.3 years). The mean duration of illness was 65.3 months (*SD* = 61.7; range 10–174 months) and the time from latest hypersomnia attack was 11. 7 months (*SD* = 22.3; range 0.5–89 months).

The participants gave written informed consent to participate according to the declaration of Helsinki. In addition, participants were provided with written information about the procedures employed in the study. The regional ethical review board in Linköping approved the study, including the consent procedure, and information materials.

All image analysis comparing brain activity and functional connectivity in healthy participants and KLS patients were performed between matched groups (18 patients and 18 controls). The analysis of behavioral data and effect sizes (see below) was performed using the whole sample.

### Experimental design

The fMRI paradigm consisted of a working memory task modeled after the Daneman and Carpenter reading span task (Daneman and Carpenter, 1980). The paradigm and the procedure are more extensively described in our previous study (Engström et al., 2009). Sentences comprising five words in Swedish were created from a list of medium frequency words. Half of the sentences were semantically correct and the other half were incorrect. The working memory task was carried out two times: first outside of the scanner and then during the scanning session (approximately 1 h after the first session). Outside of the scanner, participants were administered a “paper-and-pencil” version of the complex working memory task (listening span). Participants listened to sentences read out one by one by the investigators. Participants were instructed to respond “right” or “wrong” as soon as possible following the presentation of a sentence. Furthermore, participants were asked to remember the last word of each sentence. After a participant had given the final “right–wrong” response to two, three, four, or five successive sentences, he/she was asked to recall the final words of the sentences in correct order. Thus, the overall procedure is similar to the reading span task, with the difference of presentation modality and a free (serial) recall test instead of the recognition procedure employed in the scanner version of the tasks. Despite these differences, the tasks were intercorrelated, particularly at the most difficult levels of the task. The correlation was 0.24 between listening span and level one and two of the scanner task, and 0.61 for the more difficult levels three and four. The magnitude of these correlations was comparable to earlier reports from the literature about intercorrelations between complex working memory tasks (Shipstead et al., 2012). The results from the listening span test (the total number of correctly recalled memoranda) were used for further analyses to indicate the working memory capacity of an individual participant.

**Figure 1:**
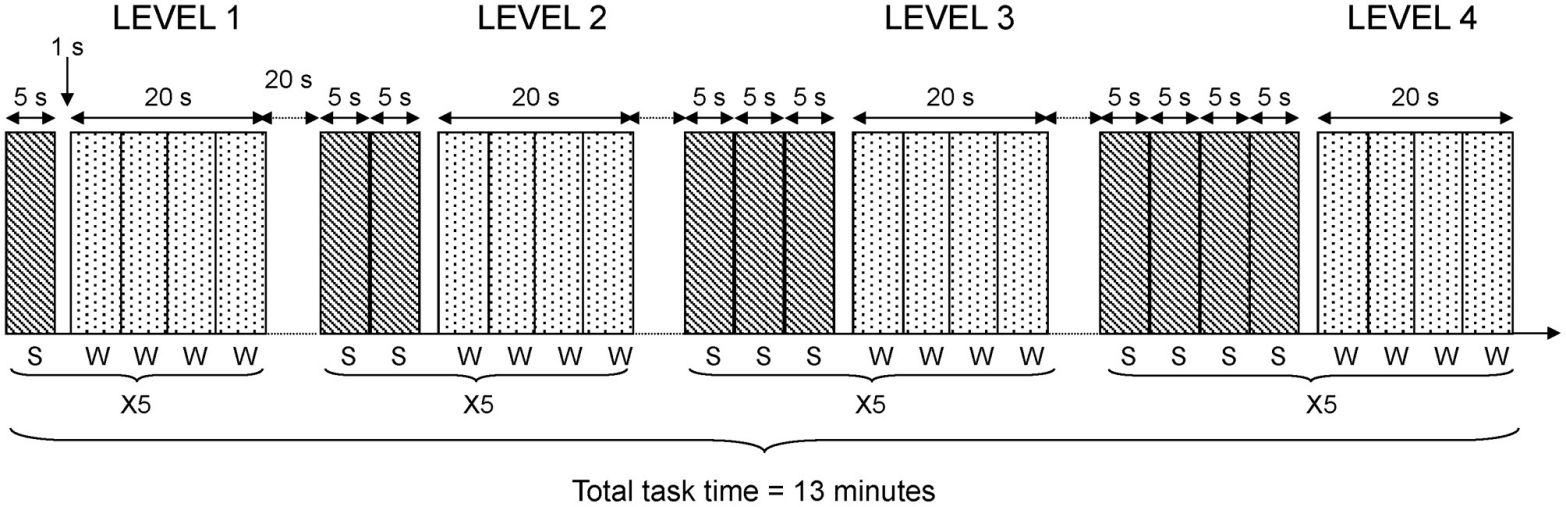
**fMRI task design.** The diagram shows details in the fMRI task design. Each level of difficulty was repeated 5 times (X5). Each sentence (S) was presented during 5 s. Each word (W) to be recognized was presented during 5 s and four words were blocked together giving 20 s blocks of word presentation in each repetition of the task.

In the fMRI-version of the task, four difficulty conditions consisting of 1–4 sentences were used. Each sentence was presented visually during 5 s, using video goggles (Resonance Technology Inc., LA, USA) and the Superlab Pro software (Cedrus Corp., San Pedro, USA). This procedure resulted in sentence blocks of 5, 10, 15, and 20 s. The four difficulty conditions were presented in the same order for all participants. The participants were instructed to answer if the sentences were correct or incorrect by pressing one of two predefined buttons on a button response box (LUMItouch, Photon Control Inc., Burnaby, Canada). It was emphasized that both speed of response and accuracy were important. Following the presentation of a varying number of sentences, a probe remained for one second. After the probe, four words (both target and lure words) were presented sequentially. Each word was presented during 5 s. Hence, the response phase always lasted 20 s. Participants were asked to indicate as quickly as possible if the word was new or old. Participants pressed their index finger to indicate targets or the middle finger for lures. This general procedure was repeated five times at each level of difficulty; that is, after one, two, three, and four consecutive sentences. The total time for the fMRI working memory task was 13 min. A diagram depicting task details in the fMRI design is shown in Figure 1.

### Procedure

The experiment comprised three parts. In the first, participants were given a paper-and-pencil version of the working memory (listening span) task (Engström et al., 2009). The second part of the experiment involved familiarization with a shortened version of the working memory task used during the MRI scan (each level of difficulty was repeated two times instead of five). If a participant failed to grasp the procedure, the familiarization trial could be repeated up to three times. However, none of the participants enrolled in this study failed to understand the procedure. The third part of the experiment took part in the MRI scanner. Different stimuli were used during all these three sessions in order to avoid learning that could contaminate working memory related processes.

### MRI

An echo planar imaging (EPI) sequence, sensitive to the BOLD response, on a Philips Achieva 1.5 T body scanner was used to acquire the functional images. The following imaging parameters were employed: *TE* = 40 ms, *TR* = 2.7 s, flip angle 90°, number of slices = 32 (with no slice gap), voxel size = 3 mm^3^, number of dynamics (volumes) = 302. The slice direction was oblique, aligned between the floor of the sella turcica and the posterior angle of the fourth ventricle according to clinical standard procedures. The slices were acquired in interleaved fashion.

### Image analysis

#### fMRI

The BOLD images were preprocessed and analyzed employing a general linear model (GLM) implemented in SPM5 software (Wellcome Department of Imaging Neuroscience, University College, London, UK). All images were realigned to correct for movement during scanning. In addition, images were normalized and resliced to MNI (Montreal Neurological Institute) template. The normalized images were smoothed with 8 mm Gaussian kernels. The BOLD time course was modeled using the canonical hemodynamic response function implemented in SPM5. Five different conditions (sentence reading and word recognition at the four different difficulty levels separated) were modeled using a block design. A high pass filter with a cut-off period of 128 s was used to remove low-frequency fluctuations.

Contrasts for word recognition after one, two, three, or four sentences (Level 1–4) were calculated employing a linear function across all four difficulty levels of memory retrieval, which was presumed to tap different levels of effort during the working memory task. Level weighting (a contrast vector of 0, −3, −1, 1, and 3) was chosen to make the weighting sum equal to zero and maintain equal interval between weights. The first column in the design matrix with contrast weight zero represented sentence reading. Random effects analyses (one-sample *t*-tests) were applied using the contrast images of each participant. In this way we obtained functional maps of each group; KLS patients and healthy controls, separately.

To depict differences between patients and controls in the executive and core networks, we applied a Region of Interest (ROI) analysis. The Wake Forrest University (WFU) PickAtlas Tool (Maldjian et al., 2004) was used to construct the ROIs in AIC (intersection of BA13 and BA47), ACC (BA32), DLPFC (BA9 and BA46), PPC (BA40), and the thalamus. As we applied a linear function to model the increasing brain activation at increasing task difficulty, we could not use a simple *t*-test to compare brain activation between groups. Instead we constructed generously thresholded functional masks for each group (mask *p*-value = 0.05). These masks were used to remove activation below this threshold (= exclusive masking) from the functional maps of the other group. In this way, the functional map of each group contained activation that was unique for the respective group. In all GLM analyses, an initial threshold of *p* = 0.001 (uncorrected) and a spatial threshold of 10 contiguous voxels were used. In this way we obtained functional maps showing activation in clusters with more than 10 voxels, however, not corrected for multiple comparisons. In order to avoid false positive results we only reported activation as significant if cluster or peak *p*-value < 0.05, corrected for multiple comparisons using the Family Wise Error (FWE) method.

In order to further assess the effect of cognitive load (or effort) on the brain activation we calculated the level-dependent effect sizes, which are mean beta regression coefficients. These are measures of the magnitude of brain activity at each level of difficulty (Levels 1–4). The effect sizes in each ROI were calculated for each participant using the MarsBar toolbox (Brett et al., 2002). However, as the effect sizes were calculated as the mean value of all voxels (activated and non-activated) in a certain region we opted to use small volumes of interest (VOI) confined within each pre-defined ROI to avoid possible zero averaging effects. The VOIs were constructed from a sphere of 10 mm centered on the healthy participant's pooled activation peak in the central executive and salience networks. In addition, a sphere in the left thalamus was constructed based on the thalamic activation peak in KLS data. The spherical VOIs were confined within the targeted anatomical regions. In order to avoid influence of activation in voxels outside the VOIs, which could be introduced by the spatial smoothing, the effect sizes were calculated from unsmoothed images.

#### Functional connectivity

One objective for the present study was to compare how interactions within and between the executive and the salience networks are modulated by cognitive effort and individual capacity. Since independent component analysis (ICA) can distinguish intrinsic connectivity from task-related connectivity (McKeown and Sejnowski, 1998; Joel et al., 2011) we chose this method to estimate the functional connectivity within and between the executive and salience networks. In this study, the Gift toolbox v1.3f (icatb.sourceforge.net) applying the Infomax algorithm was used for ICA. In order to compare the functional connectivity during an easy (presumably non-effortful) condition with the connectivity during a difficult (presumably effortful) condition, we opted to use data from the first (Level 1) and the last (Level 4) quarters in the time series acquired during performance of the working memory task. In a *post-hoc* analysis, we concluded that Level 1 could be regarded as a baseline, since no significant activation was found in ROIs except for bilateral PPC. The activation in PPC at Level 1 was found lateral to the task-related activation across all difficulty levels. Thus the PPC activation at Level 1 could be a reflection of the default mode network and accordingly similar to baseline activation. These two parts of the data-set were considered as two sessions in the analysis. We calculated 30 independent components in total. The spatial extensions of the components were evaluated across both sessions (Level 1 and Level 4). Two components representing the executive network and one component representing the salience network were selected for further analysis. In the subsequent two-sample *t*-test an initial significance threshold of *p* = 0.01 (uncorrected) and a voxel threshold of 10 contiguous voxels were used. Activation was reported as significant if cluster or peak *p*-value < 0.05, FWE corrected.

### Statistics

Analysis of behavioral results and activation levels were carried out by means of split-plot factorial (repeated measurements) ANOVAs, using IBM SPSS Statistics, version 20. When repeated measures were involved, we used Mauchley's test to make sure that the assumption of sphericity was not violated. In cases where sphericity was violated, we adjusted the degrees of freedom by means of the Greenhouse-Geisser correction.

**Figure 2:**
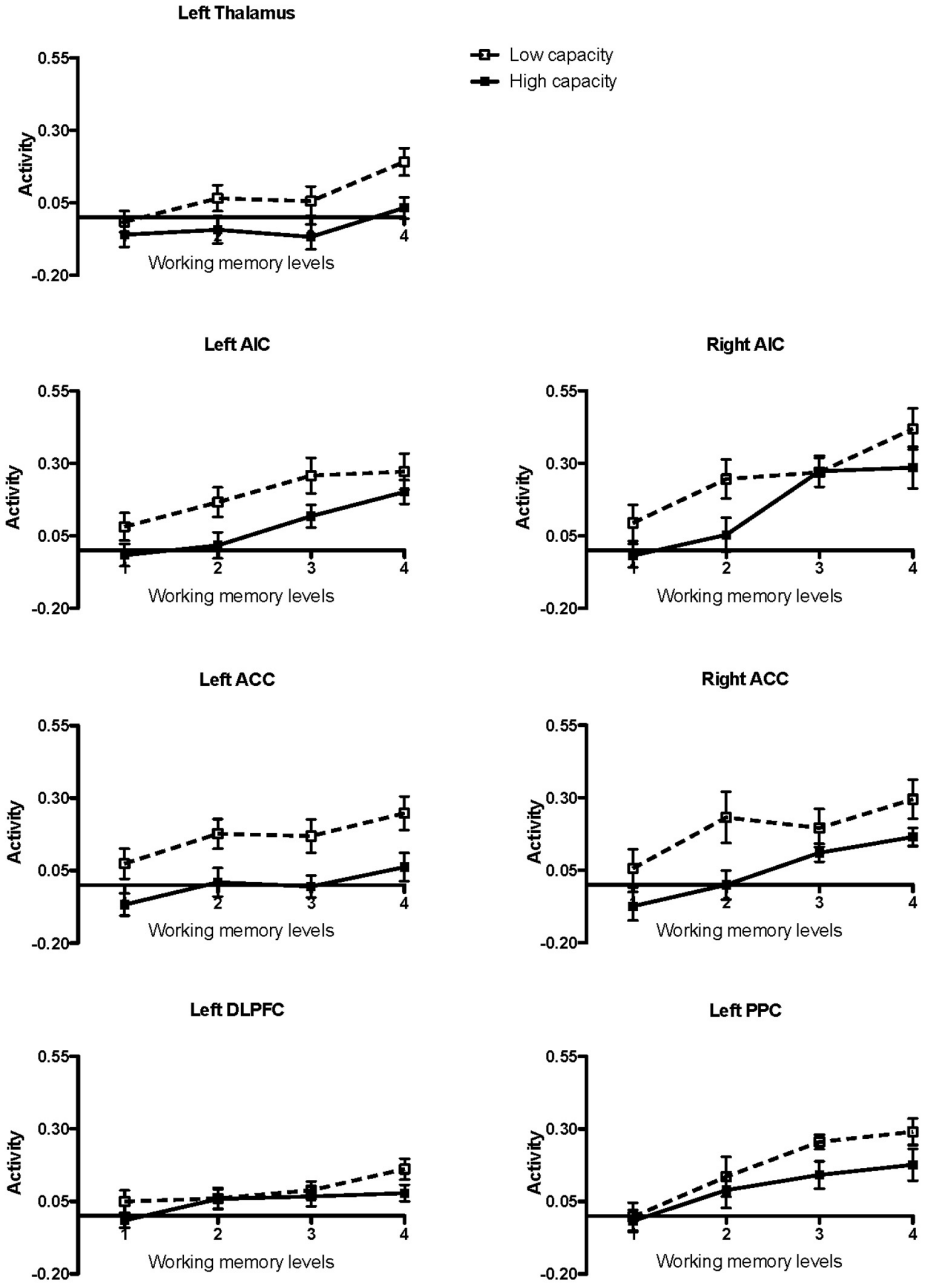
**Brain activation in low and high capacity groups at different levels of difficulty.** The graphs show the activation calculated in small volumes of interest within the pre-defined regions of interest (ROIs): AIC, Anterior insular cortex; ACC, Anterior cingulate cortex; DLPFC, Dorsolateral prefrontal cortex; PPC, Posterior parietal cortex. The participants were divided into low and high capacity groups based on a median split, mixing healthy participants and KLS. The figure depicts mean beta regression coefficients and standard error of mean (SEM).

High and low working memory capacity groups were formed by a mean split of all participants (healthy controls and KLS) according to their results on the paper-and-pencil listening span task performed before the fMRI session (mean = 18.3; *SD* = 4.9). The main reason for a categorical split of the participants into two groups was to investigate if there were any signs of different brain responses to increasing task difficulty (Figure 2). The ANOVA was accordingly carried out using dichotomization.

## Results

### Behavioral data

**Figure 3:**
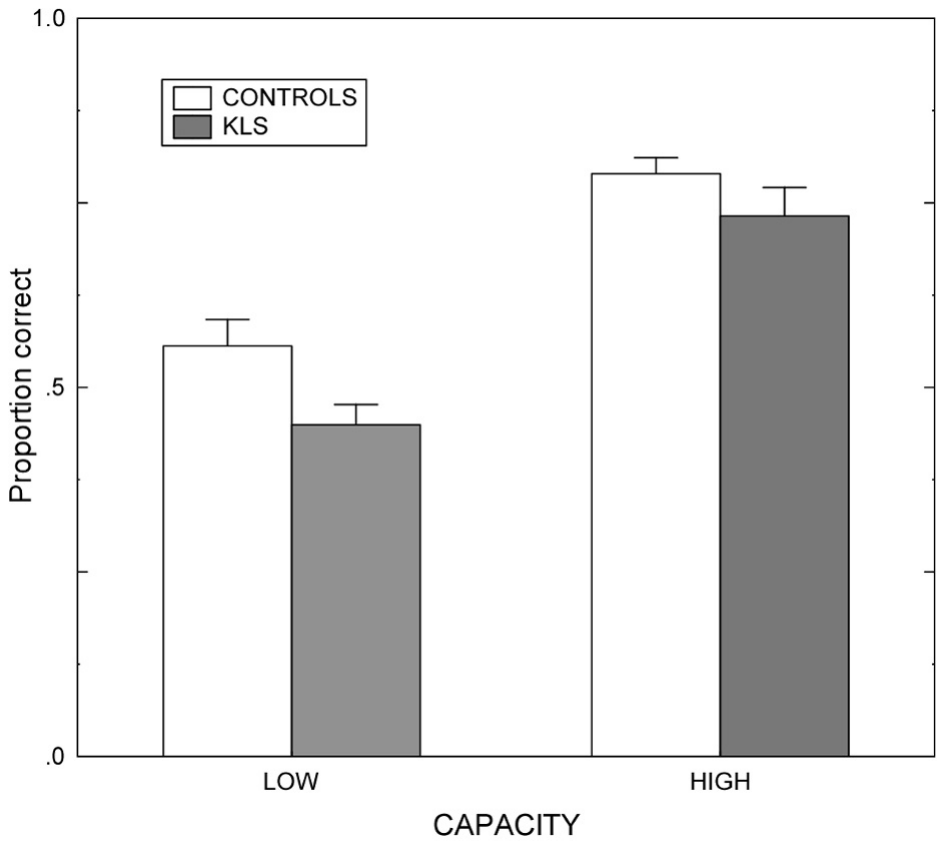
**Working memory performance.** Results from the listening span working memory tasks administered before the scanning session in healthy controls and patients with periodic idiopathic hypersomnia (Kleine–Levin syndrome, KLS). Low and High denotes high and low performance on the working memory task administered before fMRI. The graph shows the proportional mean and standard error of mean (SEM) for words correctly recalled.

Results regarding the working memory span (WMSpan) task are shown in Figure 3. The results were analyzed by means of a 2 × 2 (Group by Capacity) split-plot factorial ANOVA. (The factor Group refers to the contrast between KLS participants and healthy controls, and the factor Capacity denotes the contrast between participants of high and low working memory capacity.) The main effect of Group was statistically significant: *F*_(1, 40)_ = 6.72, *MSe* = 7.05, *p* = 0.013, η^2^_*p*_ = 0.144. This means that overall controls recognized more words than KLS participants. Also, the effect of Capacity was statistically significant; *F*_(1, 40)_ = 66.51, *MSe* = 7.05, *p* < 0.001, η^2^_*p*_ = 0.624. High-capacity participants in both KLS and the control group recognized more words than individuals of low working memory capacity. In contrast, the Group by Capacity interaction failed to influence the outcome (*F* < 1). The outcome ascertained that the independent variables (Group and Capacity) influenced performance in an additive manner. None of these findings are unexpected. Indeed, the significant main effects of Group and Capacity were prerequisites for the validity of ensuing analyses of MR data.

### Activated areas

**Figure 4:**
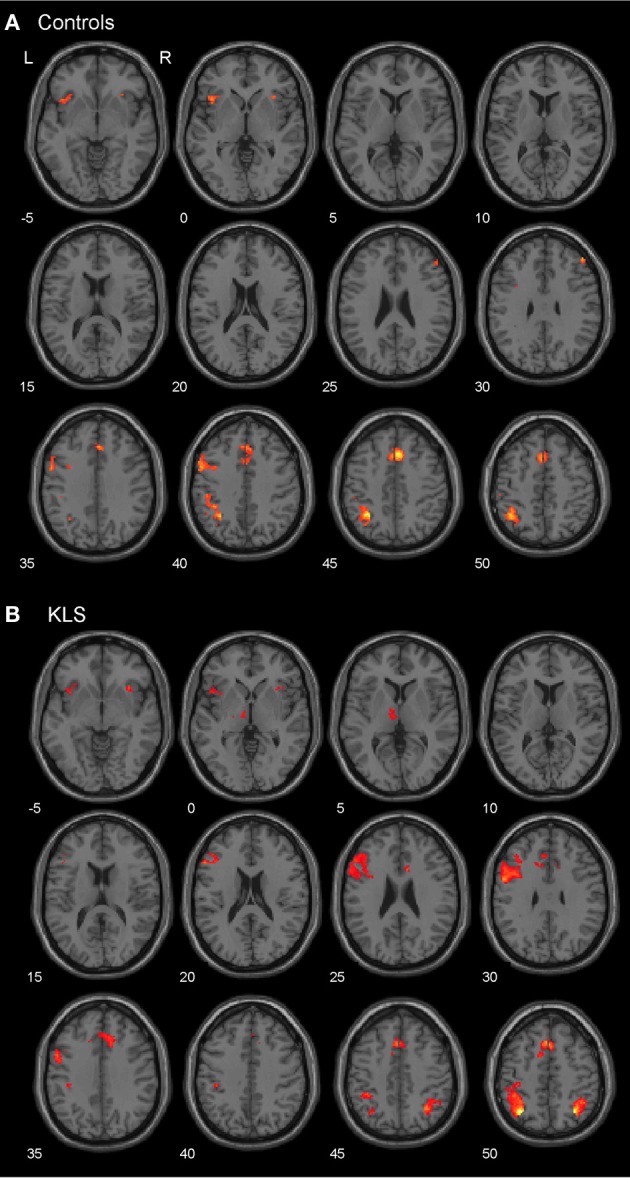
**Brain activation reflecting increasing working memory effort.** The images show brain activation in the pre-defined regions of interest (ROIs) in the executive and salience networks during the complex working memory task, which was analyzed for increasing task difficulty. **(A)** Brain activation in healthy controls. **(B)** Brain activation in patients with periodic idiopathic hypersomnia (Kleine–Levin syndrome, KLS).

As predicted, BOLD activation at increased effort in verbal working memory was found in the central executive network (DLPFC–PPC) as well as in the salience network (AIC–ACC) (Figure 4A). As shown in Figure 4B, KLS patients activated similar areas as healthy controls, but there were also some important differences, as detailed below.

#### The executive network

Figure 4B shows that KLS patients had more activation in the left DLPFC, especially in the superior parts of DLPFC. This observation was statistically significant. In the PPC, the KLS patients activated the right hemisphere more than controls, but the left hemisphere less than the controls. The difference between KLS patients and controls in the left hemispheric PPC was that the controls activated the inferior parts of PPC more than KLS.

#### The salience network

By visual inspection of Figures 4A,B, it seems that KLS patients activated the left thalamus whereas the controls did not. Indeed, in the two-sample comparison between KLS and controls it was shown that KLS patients had significantly more activation in the left thalamus (see Table 1 for significance measures and cluster sizes). There was also a dissociation involving the left and the right hemispheric ACC: KLS patients activated the right ACC more than controls and the left ACC less than the controls. Finally, the KLS patients had less activation in the left AIC, but we did not observe any significant activation difference in the right AIC.

**Table 1:** **Difference in brain activation between healthy controls and patients with periodic idiopathic hypersomnia**.

**Comparison**	**Brain area**	**BA**	***x***	***y***	***z***	**Cluster *p***	**Peak *p***	**#**
KLS > HC								
	Left DLPFC	46	−48	32	24	<0.001	<0.001	184
	Right PPC	40	34	−56	50	<0.001	<0.001	164
	Right ACC	32	12	28	36	0.020	0.021	38
	Left Thalamus	NA	−8	−10	4	<0.001	<0.001	121
KLS < HC								
	Left PPC	40	−40	−40	60	0.013	0.010	49
	Left ACC	32	−2	16	42	0.045	0.050	26
	Left AIC	NA	−36	18	−8	0.013	0.088	19

Difference in effort related working memory Blood Oxygen Level Dependent (BOLD) activation between healthy controls (HC) and patients with periodic idiopathic hypersomnia (Kleine–Levin Syndrome: KLS). The table shows brain areas and corresponding Brodmann areas (BA) in pre-defined Regions of Interest (ROIs), co-ordinates in Montreal Neurological Institute (MNI) space (x, y, z), cluster p-value (Family Wise Error, FWE, corrected), peak p-value (FWE corrected), and the number of activated voxels (#). AIC, anterior insular cortex; ACC, anterior cingulate cortex; DLPFC, dorsolateral prefrontal cortex; PPC, posterior parietal cortex.

### Activation levels

For each pre-defined ROI (i.e., the left DLPFC and PPC in the executive network and the bilateral ACC and AIC, and the left thalamus in the salience network), activation levels were analyzed by means of seven different 2 × 2 × 4 (Healthy Controls/KLS Group by Working Memory Capacity Group by Effort) split-plot factorial ANOVA:s. The two first factors were between-groups factors and Effort (with four levels of difficulty) factor was a within-groups factors. The results are summarized in Table 2.

**Table 2:** **Summary of results regarding estimates of effect size**.

**Region**	**Capacity**	**Group**							
		**HC**				**KLS**			
		**Effort**				**Effort**			
		**One**	**Two**	**Three**	**Four**	**One**	**Two**	**Three**	**Four**
ACC, L	Low	0.14	0.24	0.17	0.33	0.05	0.16	0.18	0.23
	High	−0.06	0.03	0	0.08	−0.04	−0.05	−0.01	−0.02
ACC, R	Low	0.05	0.18	0.31	0.24	0.12	0.31	0.15	0.34
	High	−0.07	0.01	0.13	0.18	−0.18	−0.04	0.04	0.14
AIC, L	Low	−0.02	0.23	0.27	0.32	0.17	0.14	0.27	0.23
	High	−0.02	0.02	0.13	0.17	−0.04	0.01	0.06	0.30
AIC, R	Low	0.08	0.32	0.35	0.56	0.15	0.25	0.24	0.34
	High	−0.01	0.05	0.24	0.25	−0.07	0	0.32	0.42
DLPFC, L	Low	0	0.11	0.11	0.25	0.10	0.04	0.09	0.10
	High	−0.04	0.04	0.04	0.07	0.05	0.08	0.11	0.12
PPC, L	Low	−0.05	0.17	0.15	0.28	0.01	0.13	0.30	0.27
	High	−0.01	0.13	0.18	0.23	−0.01	−0.08	0.04	0.07
Thalamus	Low	0.07	0.06	0.03	0.10	−0.05	0.09	0.05	0.21
	High	−0.07	−0.04	−0.06	0.01	−0.01	−0.04	0	0.18

Table entries depict mean beta regression coefficients (standard error of mean, SEM, in parenthesis). HC, Healthy controls; KLS, patients with idiopathic hypersomnia; AIC, anterior insular cortex; ACC, anterior cingulate cortex; DLPFC, dorsolateral prefrontal cortex; PPC, posterior parietal cortex.

#### The executive network

In the DLPFC, the effect of Effort was significant, *F*_(2.414, 96.60)_ = 4.576, *MSe* = 0.021, *p* = 0.008, η^2^_*p*_ = 0.103. No other main or interaction effects were close to statistical significance, except for the Effort by Group interaction, *F*_(2.414, 96.60)_ = 2.58, *MSe* = 0.021, *p* = 0.070, η^2^_*p*_ = 0.061. For the PPC, the effect of Effort was significant, *F*_(2.32, 92.81)_ = 9.417, *MSe* = 0.021, *p* < 0.001, η^2^_*p*_ = 0.191. No other main or interaction effects were statistically significant.

#### The salience network

For the left ACC, the effect of Effort was significant, *F*_(3, 120)_ = 2.74, *MSe* = 0.04, *p* = 0.046, η^2^_*p*_ = 0.060. Likewise, the effect of Capacity was significant: *F*_(1, 40)_ = 15.8, *MSe* = 0.09, *p* < 0.001, η^2^_*p*_ = 0.28. The main effect of Group was non-significant, *F*_(1, 40)_ = 1.42, *MSe* = 0.90, *p* = 0.24, η^2^_*p*_ = 0.034. All other effects displayed *F*-values smaller than unity. For the right ACC, the main effect of Effort was significant, *F*_(2.43, 97.2)_ = 5.92, *MSe* = 0.079, *p* = 0.002, η^2^_*p*_ = 0.129. The effect of Capacity was also statistically significant, *F*_(1, 40)_ = 13.42, *MSe* = 0.097, *p* = 0.001, η^2^_*p*_ = 0.251. All other main and interaction effects produced *F*-values smaller than or very close to unity.

Turning next to the left insula, the effect of Effort was significant, *F*_(2.527, 101.1)_ = 11.61, *MSe* = 0.039, *p* < 0.001, η^2^_*p*_ = 0.225. The effect of Capacity was also significant: *F*_(1, 40)_ = 5.46, *MSe* = 0.104, *p* = 0.025, η^2^_*p*_ = 0.120. In addition, the Effort by Group by Capacity three-way interaction produced a marginally significant effect, *F*_(2.527, 101.063)_ = 2.45, *MSe* = 0.039, *p* = 0.078, η^2^_*p*_ = 0.058. No other effect approached statistical significance. Regarding the right insula, the effect of Effort was significant, *F*_(3, 120)_ = 21.89, *MSe* = 0.041, *p* < 0.001, η^2^_*p*_ = 0.354. The effect of Capacity was marginally significant: *F*_(1, 40)_ = 3.66, *MSe* = 0.187, *p* = 0.063, η^2^_*p*_ = 0.084. Also, the Effort by Capacity interaction was marginally significant, *F*_(3, 120)_ = 2.34, *MSe* = 0.041, *p* = 0.077, η^2^_*p*_ = 0.055. More important, the Effort by Group by Capacity three-way interaction was significant, *F*_(3, 120)_ = 2.94, *MSe* = 0.041, *p* = 0.036, η^2^_*p*_ = 0.069. Using Bonferroni-corrected comparisons of the cell means and employing a *p*-value < 0.05, the means at the lowest level of effort for controls in the high performance group were different from means for the low-capacity KLS group. While this arguably is a conservative approach, the result reflects the tendency for particularly high working memory controls to produce lower beta coefficients.

Finally, for the thalamus the main effect of Effort was significant, *F*_(3, 120)_ = 5.704, *MSe* = 0.028, *p* = 0.001, η^2^_*p*_ = 0.125. No other main or interaction effects were statistically significant.

It can be seen from Table 2 that Effort modulated the activity at all ROI:s. More interestingly, working memory capacity produced effects in the salience network (AIC and ACC). In the left hemisphere, this difference appeared as a statistically significant main effect of the Working Memory Capacity Group factor, indicating an overall difference between participants with high or low working memory capacity. A concern with dichotomizing working memory is that this procedure may produce spurious effects, sometimes exaggerating effects of a dichotomized continuous variable, at other occasions producing a drop in statistical power (MacCallum et al., 2002). Hence, we also took a hierarchical approach to our data, employing the MIXED procedure of IBM SPSS. Since Effort was a repeated measure, we used a first-order autoregressive covariance structure to model the repeated measure (which was nested under participant), and maximum likelihood estimation. For all regions of interest, a model including effects of Effort, Group, and WMSpan (the performance on the paper-and-pencil listening span task which was dichotomized into the Capacity variable in the ANOVAs above) produced an optimal fit of the model. Adding two and three-way interactions did not give rise to a model statistically better than the main-effects model (in terms of −2 log likelihood).

For the left ACC, the effects of Effort was significant, *F*_(1, 148.99)_ = 6.37, *p* = 0.013. Also, the effect of WMSpan was significant, *F*_(1, 64.9)_ = 7.42, *p* = 0.008. For the right ACC, Effort was significant, *F*_(1, 141.9)_ = 10.1, *p* = 002; as was WMSpan, *F*_(1, 50.9)_ = 4.26, *p* = 044. For the left insula, only Effort produced a significant effect, *F*_(1, 46.52)_ = 20.4, *p* < 0.001. A similar pattern emerged for the right insula in that only Effort was statistically significant, *F*_(1, 145.6)_ = 41.1, *p* < 0.001. Similar effects of Effort emerged for DLPFC, *F*_(1, 56.9)_ = 10.8, *p* = 0.012; PPC, *F*_(1, 147.8)_ = 28.0, *p* < 0.001; and the thalamus, *F*_(1, 149.0)_ = 13.4, *p* < 0.001. Thus, the two approaches to analyzing the effects of working memory effort and capacity produced similar results, with the exception of the left insula, where the repeated measures ANOVA indicated an effect of capacity, specifically.

**Figure 5:**
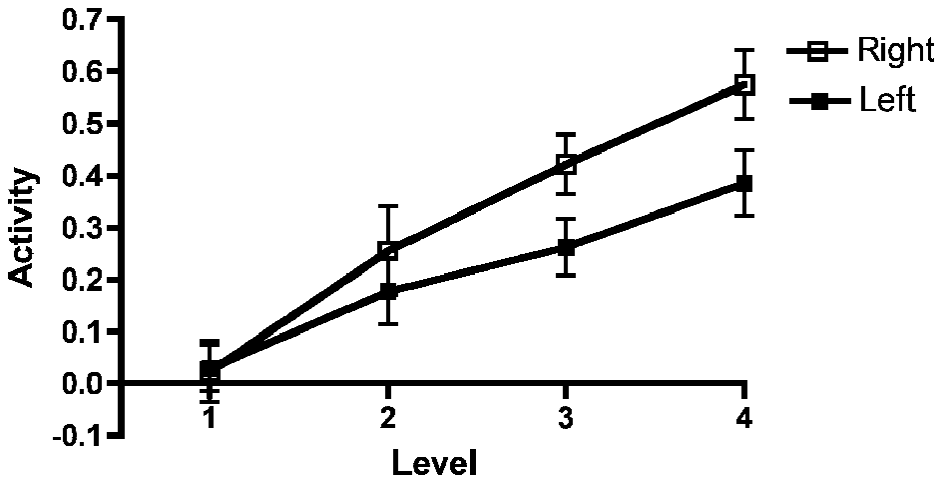
**Left and right AIC/AAC activations as a function of effort.** Results for the left and right anterior insular cortex (AIC)/anterior cingulate cortex (ACC) regions of interest (ROI) combined across all participants as a function of effort. The figure depicts mean beta regression coefficients (mean value and SEM).

Upon inspection of the results, we noted a steeper increase of the BOLD signal in the right AIC/ACC than in the left counterparts. This pattern can be seen in Figure 5. To confirm this impression, we carried out an additional ANOVA adding Region (i.e., AIC vs. ACC) and Hemisphere (Right vs. Left) to the previously described Group, Working Memory Capacity, and Effort factors. As before, there were significant effects of Capacity [*F*_(1, 40)_ = 10.3, *MSe* = 0.031, *p* = 0.003, η^2^_*p*_ = 0.204] and Effort [*F*_(3, 120)_ = 25.5, *MSe* = 0.078, *p* < 0.0001, η^2^_*p*_ = 0.389]. There was also a significant main effect of Hemisphere: *F*_(1, 40)_ = 8.00, *MSe* = 0.191, *p* = 0.007, η^2^_*p*_ = 0.167. Finally, there was a significant Effort by Hemisphere interaction: *F*_(3, 120)_ = 5.4, *MSe* = 0.043, *p* = 0.002, η^2^_*p*_ = 0.120. No other effect reached conventional criteria for statistical significance, although the Effort by Working Memory Capacity by Hemisphere three-way interaction approached statistical significance; *F*_(3, 120)_ = 2.27, *MSe* = 0.043, *p* = 0.084, η^2^_*p*_ = 0.056. Multi-level modeling (with hemisphere and Effort nested under participant; see also above for general details about the calculation) indicated that the optimal model involved effects of WMSpan, *F*_(1, 101.2)_ = 14.0, *p* < 0.000; Region, *F*_(1, 656.5)_ = 16.1, *p* < 0.001; and the Effort by Hemisphere interaction, *F*_(1, 543.2)_ = 71.5, *p* < 0.001. However, it should be noted that this model did not produce a significantly better fit than a model involving only the main effects, χ^2^_(3)_ = 5.16, *p* = 0.160. In this latter model, Hemisphere, *F*_(1, 659.5)_ = 9.71, *p* < 0.001; and Effort, *F*_(1, 276.7)_ = 68.1, *p* < 0.001, were significant in addition to WMSpan [*F*_(1, 104.5)_ = 11.7, *p* = 0.001] and Region [*F*_(1, 659.5)_ = 16.2, *p* < 0.001].

### Functional connectivity

Three independent components obtained from the conjoint analysis of the low (Level 1) and high (Level 4) effort levels, were selected for further analysis. One component represented a mainly left lateralized network encompassing the ventrolateral and DLPFC and the temporal lobe in the left hemisphere as well as the bilateral parietal cortex. The second component represented substantially the right-sided homologue to the first component. These two components included the executive network defined as DLPFC and PPC. Finally, the third component encompassed mainly the bilateral insulae, ACC, and parts of the temporal lobe thus representing the salience network. The thalamus was also included in the salience network according to the ICA. The right thalamus was significantly coupled to the network (cluster *p* = 0.012). However, couplings between the salience network and the left thalamus fell just below the selected threshold (cluster *p* = 0.059). Supplementary figures of the independent components representing the executive and the salience networks can be obtained from the corresponding author.

#### Executive network

**Figure 6:**
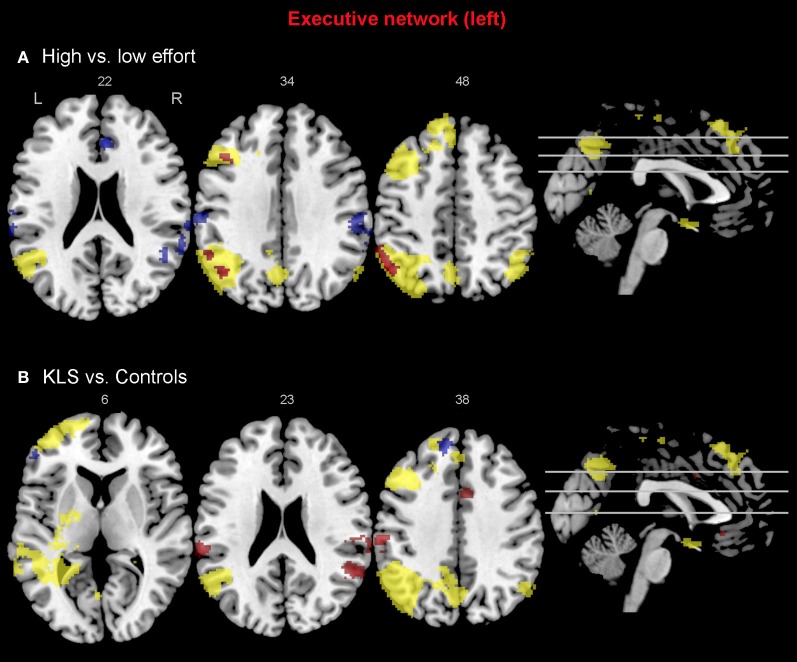
**Functional connectivity in the left-hemispheric executive network.** The images show the results from the independent component representing the left-hemispheric executive network. Yellow areas show the executive network in selected slices (significance threshold: *p* = 0.001). Red and blue areas show connectivity differences in regions of interest (ROIs). Connectivity difference images were thresholded at *p* = 0.05 for visualization purpose. **(A)** Red color denotes regions that are more strongly coupled to the network at the high effort level (Level 4) compared to the low effort level (Level 1). Blue color denotes regions that are more strongly coupled to the network at the low effort level. **(B)** Regions that are more strongly (red) respectively more weakly (blue) coupled to the network in patients with periodic idiopathic hypersomnia (KLS) compared to healthy participants at the high effort level.

**Figure 7:**
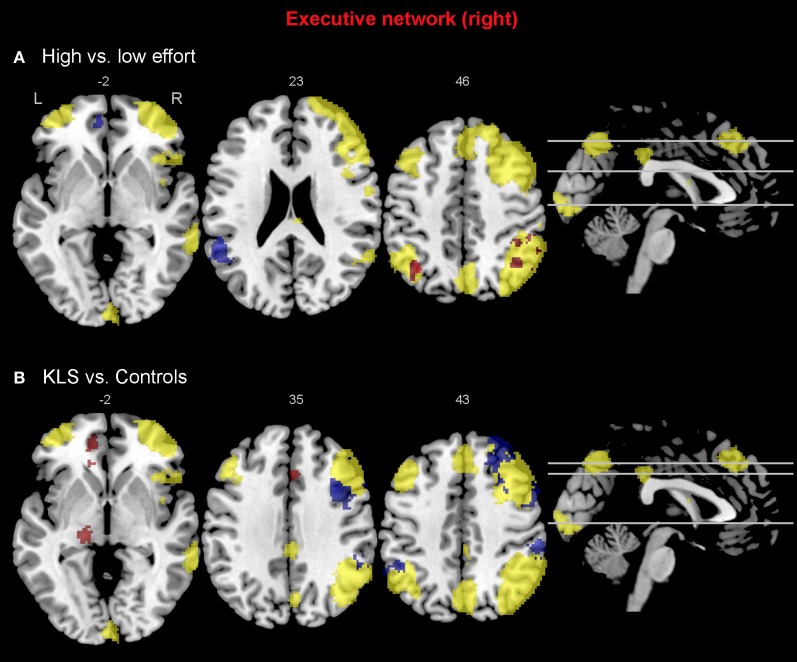
**Functional connectivity in the right-hemispheric executive network.** The images show the results from the independent component representing the right-hemispheric executive network. Yellow areas show the network in selected slices (significance threshold: *p* = 0.001). Red and blue areas show connectivity differences in regions of interest (ROIs). Connectivity difference images were thresholded at *p* = 0.05 for visualization purpose. **(A)** Red color denotes regions that are more strongly coupled to the network at the high effort level (Level 4) compared to the low effort level (Level 1). Blue color denotes regions that are more strongly coupled to the network at the low effort level. **(B)** Regions that are more strongly (red), respectively, more weakly (blue) coupled to the network in patients with periodic idiopathic hypersomnia (KLS) compared to healthy participants at the high effort level.

When comparing the high and low effort levels of the independent components representing the executive network for both groups, we found that there were stronger couplings within the network at the high effort level. As can be seen in Figures 6A, 7A, all areas that were significantly stronger coupled to the network at the high effort level compared to the low effort level (red color) were located within the network (yellow color). Significance measures and cluster sizes are given in Table 3. Specifically, at the high effort level, there were stronger couplings between the left-hemispheric executive network and regions in the left DLPFC and the left PPC (Figure 6A). For the right-hemispheric executive network, there were stronger couplings within bilateral parietal regions of the network, at the high effort level compared to the low effort level (Figure 7A). In contrast, at the low effort level there were stronger couplings to parietal regions beyond both the left and the right-sided components of the executive network (blue color in Figures 6A, 7A). At the low effort level there were also stronger couplings between the executive network and ACC in the contra-lateral hemisphere, i.e., between the left-sided component and the right ACC and between the right-sided component and the left ACC.

**Table 3:** **Difference in functional connectivity between high and low effort in the left executive, the right executive, and the salience networks during execution of the working memory task**.

**Network**	**Effort**	**Region**	**BA**	***x***	***y***	***z***	**Cluster *p***	**Peak *p***	**#**
Left executive	High > Low	Left DLPFC	46	−40	22	28	0.046	0.038	17
		Left PPC	40	−50	−62	44	0.018	0.037	38
	Low > High	Left PPC	40	−62	−20	28	0.018	0.018	37
		Right PPC	40	62	−46	24	0.033	0.011	24
		Right PPC	40	52	−26	38	0.008	0.016	58
		Right PPC	40	32	−38	40	0.044	0.017	18
		Right ACC	32	4	32	22	0.036	0.028	22
Right executive	High > Low	Left PPC	40	−34	−64	48	0.009	0.009	54
		Right PPC	40	46	−34	38	0.026	0.059	29
	Low > High	Left PPC	40	−26	−50	52	0.016	0.012	40
		Left PPC	40	−54	−44	22	0.036	0.043	22
		Left ACC	32	−6	50	−6	0.041	0.066	19
Salience	High > Low	Left Thalamus	NA	−14	−26	8	0.046	0.052	16
	Low > High		−	−	−	−	−	−	−

The table shows brain areas and corresponding Brodmann areas (BA) in regions of interest (ROIs), co-ordinates in Montreal Neurological Institute (MNI) space (x, y, z), cluster p-value (Family Wise Error, FWE, corrected), peak p-value (FWE corrected), and the number of activated voxels (#). ACC, anterior cingulate cortex; DLPFC, dorsolateral prefrontal cortex; PPC, posterior parietal cortex.

Compared to healthy participants, KLS patients had stronger couplings between the executive network and ACC and parietal areas outside the network at the high effort level (Figure 6B). In addition, KLS patients had stronger couplings between the right-sided component of the executive network and the left thalamus. On the other hand, KLS patients had weaker couplings to within-network regions in DLPFC and PPC (Table 4). As can be seen in Figures 6B, 7B, all areas (blue color) that are more weakly coupled to the network in KLS (or more strongly coupled in controls) are located in, or adjacent to, the executive network.

**Table 4:** **Difference in functional connectivity between patients with periodic idiopathic hypersomnia (KLS) and healthy controls (HC) during the difficult level of working memory retrieval**.

**Network**	**Group**	**Region**	**BA**	***x***	***y***	***z***	**Cluster *p***	**Peak *p***	**#**
Left executive	KLS < HC	Left DLPFC		−58	24	12	0.027	0.025	26
	KLS > HC	Left PPC	40	−60	−30	28	0.030	0.010	24
		Right PPC	40	58	−48	32	0.026	0.010	27
		Left ACC	32	−4	46	−2	0.036	0.031	21
		Right ACC	32	6	32	−12	0.044	0.066	17
Right executive	KLS < HC	Right DLPFC	9	24	34	40	0.024	0.054	29
		Right DLPFC	9	28	18	42	0.036	0.056	21
		Right DLPFC	46	34	16	32	0.021	0.028	31
		Left PPC	40	−34	−52	52	0.036	0.007	21
		Right PPC	40	58	−28	48	0.010	0.007	49
	KLS > HC	Left ACC	32	−10	46	20	0.029	0.024	25
		Left ACC	32	−10	54	4	0.017	0.006	36
		Right ACC	32	22	40	4	0.020	0.022	33
		Right ACC	32	4	26	30	0.047	0.041	16
		Left Thalamus	NA	−16	−26	0	0.042	0.058	18
Salience	KLS < HC	Left DLPFC	9	−44	24	42	0.026	0.023	27
		Left DLPFC	46	−50	38	14	0.019	0.025	33
	KLS > HC	Left Thalamus	NA	−18	−22	6	0.056	0.044	13

The table shows brain areas and corresponding Brodmann areas (BA) in Regions of Interest (ROIs), co-ordinates in Montreal Neurological Institute (MNI) space (x, y, z), cluster p-value (Family Wise Error, FWE, corrected), peak p-value (FWE corrected), and the number of activated voxels (#). ACC, anterior cingulate cortex; DLPFC, dorsolateral prefrontal cortex; PPC, posterior parietal cortex.

#### Salience network

**Figure 8:**
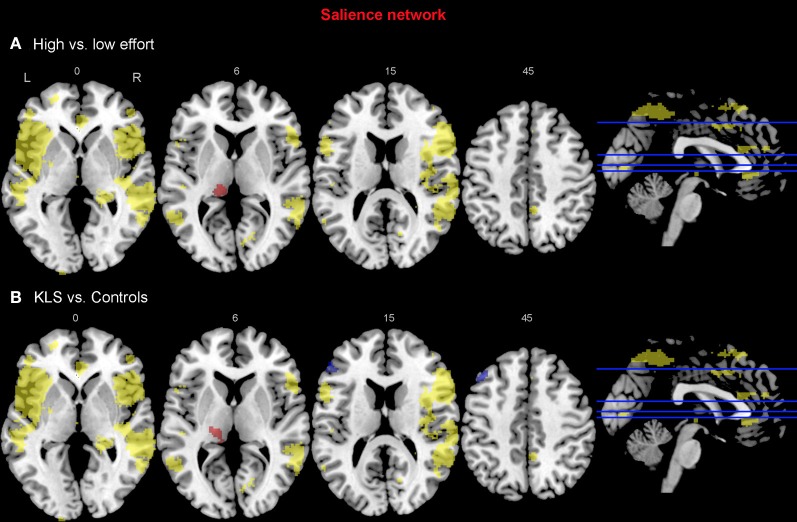
**Functional connectivity in the salience network.** The images show the results from the independent component representing the salience network. Yellow areas show the network in selected slices (significance threshold: *p* = 0.001). Red and blue areas show connectivity differences in regions of interest (ROIs). Connectivity difference images were thresholded at *p* = 0.05 for visualization purpose. **(A)** Red color denotes regions that are more strongly coupled to the network at the high effort level (Level 4) compared to the low effort level (Level 1). Blue color denotes regions that are more strongly coupled to the network at the low effort level. **(B)** Regions that are more strongly (red) respectively more weakly (blue) coupled to the network in patients with periodic idiopathic hypersomnia (KLS) compared to healthy participants at the high effort level.

For the salience network, we observed a stronger coupling to the left thalamus at the high effort level for both groups (Figure 8A, Table 3). However, KLS patients had stronger couplings to the left thalamus at the high effort level compared to the healthy controls. These findings might signify that the KLS patients drive the results of the high–low effort comparison. We also observed that the controls had stronger couplings between the salience network and the left DLPFC outside the salience network but within the executive network (Figure 8B, Table 4). Thus, for the salience network, but not for the executive network, the controls had stronger couplings outside the network.

## Discussion

There were three main findings from this study. First, and perhaps less surprisingly, complex working memory (the reading span task) produced effort-related activation of the executive network, typically involved in working memory, and in the salience network (Cabeza and Nyberg, 2000; Naghavi and Nyberg, 2005; Champod and Petrides, 2010). Second, increased capacity in the working memory task modulated the activation of the salience network. Third, high-capacity participants differed from low-capacity participants in that they evidenced lower activation in the ACC and AIC. Finally, the connectivity analysis hinted at a functional distinction between low and high effort levels as well as between healthy participants and KLS. In particular, high effort narrowed the within-network connectivity of the executive network but increasingly involved thalamic activity in the salience network.

Tasks involving complex working memory highlight the combined temporary storage and manipulation of to-be-remembered information. Such tasks include reading span and listening span (which were used in this study; reading span in the MR experiment and listening span for pre-experimental assessment), operation span (Kane et al., 2004), the n-back task (Owen et al., 2005), the Miyake spatial span (Miyake et al., 2001), and the recently introduced rule working memory task (Duncan et al., 2012). A hallmark of these tasks is that they are able to predict performance in a large number of tasks that reflect thinking, problem solving and comprehension. Although “simple” working memory tasks also do so to some extent, a key finding in recent work on attention and working memory is that complex tasks can account for substantial portions of the variance in problem solving capacity and intelligence. At present, there is no single answer as to why this association takes place. Two mechanisms have been suggested to account for this association. Complex working memory requires both online storage and active retrieval of memoranda on the one hand and executive skills, such as updating and task switching, on the other hand. Furthermore, attentional capacity may determine both online storage/retrieval and executive processing. While the critical neuroimaging experiments necessary to clarify these issues still are wanting, our results clearly would fit well with the view emphasizing attentional capacity in working memory. More specifically, although both areas and network hubs related to the executive system (the DLPFC and the PPC) and the salience network were modulated by task difficulty, only the ACC and the AIC hinted at differences between high- and low performers.

### The executive network

Working memory, including its neural substrates, is a limited-capacity system, which means that it is highly sensitive to cognitive load (Honey et al., 2002). Several authors have studied the parametric brain response to working memory in the executive network using the n-back task. Some previous studies have found a linear, capacity unconstrained, relationship between brain activity and increasing task difficulty (Jansma et al., 2007; Braver et al., 1997). However, other studies have found capacity constrained relations where the signal first peaks and then declines when the task becomes very difficult (Callicott et al., 1999). Nyberg and coworkers (2009) found a capacity unconstrained response in the executive network and also in the thalamus for high performing subjects, but a capacity constrained response for low performing subjects. This finding indicates that the brain response is modulated by individual ability. In the current study, we found a linear response to increasing task difficulty in all ROIs at the group level, as estimated by a *post-hoc* linear regression test. This overall linear response to task difficulty justifies the linear model used in the image analysis. However, results in Figure 2 indicate that individual responses to task difficulty can vary. Nevertheless, we did not find any effects of capacity in the executive network (Table 2). As can be seen in Figure 2 there is no difference between low- and high-capacity participants in DLPFC. However, the overall effect size is rather low, which could be due to a non-optimal choice of ROI or that working memory in DLPFC is divided into several interacting subregions. In PPC there seem to be a trend of capacity dependency for the higher effort levels and also a trend of a capacity constrained response for low performing subjects.

In the functional connectivity analysis, the executive network was divided into two components that essentially represented the left and the right hemisphere, respectively. The main finding was that increased effort in the working memory task generated stronger couplings within the network and weaker couplings beyond the network (Figures 6A, 7A). This pattern was also an effect of working memory capacity, in that KLS patients had weaker couplings within the network, especially in the right hemisphere, and stronger couplings beyond the network in PPC, ACC, and the left thalamus. This might indicate that KLS patients were not able to focus their mental resources to the executive network when the working memory task was made more difficult.

It is of interest to note that two recent studies have implicated a similar pattern in the executive network. Using graph theoretical tools to describe connectivity, Ginestet and Simmons (2011) noted that the weighted cost of the network (i.e., the inverse of network efficiency) decreased as difficulty increased in the n-back task. That is to say, spontaneous correlations diminished when the working memory task became more challenging. In addition, the network cost predicted the individual participants' capacity. More recently, Bluhm and coworkers (2011) investigated connectivity in specific executive network hubs. Similar to our findings, difficulty increased intrinsic connections.

In contrast to these findings, Honey and associates (2002) observed greater cross-hemispheric connectivity when comparing 2-back with 1-back tasks. This finding is seemingly contradicted by the results in our study as we find stronger cross-hemispheric connections at the low effort level. On the other hand, at the high effort level we observed stronger frontoparietal couplings in the right hemisphere, which could be a marker of greater cross-hemispheric interactions since the verbal working memory task is predominantly left lateralized.

### The salience network

With the use of a complex working memory task, we also found that effort produced activation in AIC and ACC. A main finding of this study is therefore that the salience network is highly sensitive to cognitive effort and increasing load in working memory. A few previous studies report activation of the AIC or the adjacent frontal operculum (BA 47) and the ACC during effort-demanding, load-dependent working memory tasks (Barch et al., 1997; Jansma et al., 2007; Engström et al., 2009). Co-activation of AIC and ACC was first observed during presentation of salient sensory stimuli (Downar et al., 2002) and later during a multitude of cognitive and sensory tasks (for overviews see Craig, 2009; Medford and Critchley, 2010). The salience network shows sustained activity across different task sets and is also responsive to homeostatic signals, such as pain and body temperature (Dosenbach et al., 2006; Cole and Schneider, 2007; Seeley et al., 2007; Craig, 2010; Menon and Uddin, 2010). Since signals from the salience network, in particular AIC, precedes signals from the executive network it has been proposed that AIC–ACC constitute a core network that detects salient events and regulates higher-order processes by facilitating switching between different brain nodes (Dosenbach et al., 2006; Sridharan et al., 2008; Craig, 2010; Menon and Uddin, 2010).

An obvious—and very important—implication of our finding that the salience network is highly sensitive to effort and increasing load would also be that this network plays an integral role in those functions where working memory capacity is of interest. Since working memory is related to intelligence, reading skills, and other cognitive activities, it is possible that the salience network also mediates these functions. Although this question clearly needs further attention, it is interesting that inspection time, a psychometric measure that is related to intelligence, also is related to activity in the AIC and ACC (Deary et al., 2004). Hence, it is possible that working memory capacity, intelligence, and perceptual proficiency all are related and in turn contingent upon activity in the salience network.

To further the understanding of this issue, we made a split of the participants into two groups, based upon working memory (i.e., performance in the listening span task administered outside of the scanner). Working memory capacity modulated the activity in the salience network, but not in the executive network. In the left hemisphere portions of the salience network, capacity turned out as a main effect. This was also true as to the right ACC. However, in the right hemisphere AIC there was a more complex Group by Capacity by Effort interaction. A trend of a capacity constrained response for low performing subjects was observed in the left AIC whereas no such trend was observed in ACC. According to the discussion above, such dichotomy has been reported across studies, however in the executive network (Braver et al., 1997; Callicott et al., 1999; Jansma et al., 2007; Nyberg et al., 2009).

The BOLD signal estimates were modulated by effort at most ROIs. However, there emerged two notable exceptions to this pattern, indicative of differences between high- and low performers. In the left AIC and ACC (both right and left), high performers evidenced a lower intercept. Hence, one of the reasons why high performers perform better could be because they possess a larger reservoir of neural spare capacity of the salience network, allowing them two utilize neural resources along a broader dynamic spectrum [for a recent review of neural efficacy in relation to intelligence, see Neubauer and Fink (2009)]. A similar result, pertaining to measures of pupil dilatation, was reported by Heitz and coworkers (2008). These experimenters found that pupil dilation, which often is considered as a reliable indicator of physiological effort, was larger for low-span participants and increased as a function of difficulty. Currently, we are not aware of an fMRI study directly relating BOLD estimates to pupillometric data or other physiological indicators of cognitive effort; such an experiment would undoubtedly be of great interest.

Besides the core function of the AIC–ACC network, the thalamus plays an integral role in coordinating brain function trough several thalamocortical circuits. At a functional level, behavioral studies (involving animal lesion studies or neuropsychological patients) highlight the importance of thalamic nuclei for regulation of attention and effort (Castaigne et al., 1981; Van Der Werf et al., 2003; Schiff, 2008; De Witte et al., 2011; Ward, 2011). Previous investigations (primarily involving connectivity at rest) have produced somewhat conflicting results as to the role of the thalamus in the salience network (e.g., Seeley et al., 2007). Although it remains an open question whether the thalamus fits in to the salience network or not, it is possible that thalamic involvement occurs as a function of recruitment of attentional resources; if an experiment explicitly requires recruitment of attentional resources, the thalamus will be involved, if an experiment comprises easy tasks or is designed to study resting state neural activity, thalamic recruitment can vary considerably.

### Effects of working memory deficits on the executive and salience networks

In a previous study, we showed decreased effort-related activation in the salience network and increased activation in parts of the executive network in patients with KLS and manifested working memory deficits (Engström et al., 2009). However, conclusions from that study were hampered by the very limited number of KLS participants (*n* = 8) available to us at that time. It was also the first fMRI study of KLS. Since KLS (and other forms of hypersomnia) has become an area of impending clinical and research concern, we deemed it imperative to replicate and extend our previous finding. In our previous study, the KLS patients also showed increased activation in the left thalamus. In the current study, we replicated those main findings in a larger group of participants. Most importantly, the thalamic hyperactivity in KLS patients was replicated in a new cohort. In addition, we could replicate the findings of decreased effort-related activity in AIC in the patient group.

The activation in ACC was functionally dissociated in patients with periodic idiopathic hypersomnia and in healthy controls: the patients had more activation in the right ACC whereas the controls had more activation in the left ACC. Similar left–right dissociations were found in the PPC. All in all, patients suffering from an illness (periodic idiopathic hypersomnia) that comes with long-lasting working memory deficits in a large majority of cases evidenced marked alterations in distinct parts of the executive and salience networks.

The cardinal finding from the conventional model-based analysis of BOLD data was the functional aberration in the thalamus in KLS. This finding was also prominent in the model free analysis of functional connectivity. Patients with periodic idiopathic hypersomnia evidenced stronger couplings to the left thalamus from both the executive and salience networks. Malfunction of the thalamus is possibly involved in hypersomnia since the thalamus is part of the ascending arousal system that regulates wakefulness, which also includes pathways between nuclei in the brain stem, the hypothalamus, and the cortex that regulates wakefulness (Saper et al., 2005). It has also occasionally been reported that lesions in the thalamus cause symptoms similar to periodic idiopathic hypersomnia (Guilleminault et al., 1993; Bassetti et al., 1996; De Witte et al., 2011). The present findings thus strengthen the case for involvement of thalamic structures in the etiology of hypersomnia.

## Conclusion

This communication singles out a network of neural resources that are involved in a verbal, complex working memory task. In particular, cognitive effort was related to increased activation in the salience network, in addition to the executive network typically engaged in complex working memory tasks. Low- and high-capacity participants showed an increase in activity as a function of increasing demands but differed in that high-capacity participants started from a lower level. The results suggest that the brain comprises a core control network that differentiates high- from low performers. This core network presumably provides the cognitive resources that supplement other cognitive networks in demanding tasks and situations.

### Conflict of interest statement

The authors declare that the research was conducted in the absence of any commercial or financial relationships that could be construed as a potential conflict of interest.
